# Our experience with liver and spleen elastography in the prediction of oesophageal varices

**DOI:** 10.4102/sajr.v28i1.2724

**Published:** 2024-01-25

**Authors:** Shivali Arya, Rashmi Dixit, Sneha Harish C, Anjali Prakash, Amarender S. Puri

**Affiliations:** 1 Department of Radiodiagnosis, Maulana Azad Medical College & Associated Lok Nayak Hospital, New Delhi, India; 2 Department of Radiodiagnosis, Government Medical College, Chandigarh, India; 3 Department of Gastroenterology, Govind Ballabh Pant Hospital, New Delhi, India

**Keywords:** oesophageal varices, acoustic radiation force imaging, chronic liver disease, shear wave velocity, splenic stiffness, hepatic stiffness

## Abstract

**Background:**

Variceal bleeding is an important cause of mortality in patients with chronic liver disease (CLD). The gold standard for detection and grading of oesophageal varices (EV) is upper gastrointestinal endoscopy. However, it is expensive, time-consuming and invasive.

**Objectives:**

This study aimed to find any association between splenic shear wave velocity (SWV) measured by acoustic radiation force imaging (ARFI) and the presence of EV.

**Method:**

The quasi-experimental study included 50 patients with CLD and 50 subjects without CLD as the control group. Both underwent upper abdominal ultrasonography followed by elastographic assessment on a Siemens Acuson S2000^TM^ ultrasound system. A comparison of the findings was made between the control and patient groups.

**Results:**

Both groups had similar hepatic size while patients with CLD had larger splenic size and area (*p* < 0.05). The CLD patients had higher mean hepatic and splenic SWV compared with the control group (*p* < 0.05). The mean splenic size and splenic SWV were higher in patients with varices than in those without varices (*p* < 0.05).

**Conclusion:**

Chronic liver disease causes significant increase in liver and splenic stiffness with splenic SWV values being higher for patients with varices emphasising the role of elastography as a non-invasive predictor for the presence of EVs. Splenic SWV had the highest sensitivity and specificity, which was augmented by a combination of hepatic and splenic SWV. Thus, splenic SWV alone or in combination with hepatic SWV is a useful technique for prediction of the presence of EVs.

**Contribution:**

This study aims to find an alternative non-invasive and cost-effective technique for screening of EV.

## Introduction

Chronic liver disease (CLD) in due course results in the development of liver fibrosis and portal hypertension. Oesophageal varices (EV) develop in 30% – 70% of patients with portal hypertension. Variceal bleeding is a life-threatening event with a mortality of approximately 10% – 20% per bleeding episode and a 1-year survival of about 63%.^1,2,3,4^ The gold standard for detection and grading of EV is upper gastrointestinal endoscopy (UGIE). Current guidelines require patients in a compensated stage of liver cirrhosis to undergo endoscopy to screen for EV.^5^ However, it is expensive, time-consuming and invasive. Hence, efforts have been made to find an alternative non-invasive and cost-effective technique for screening of EV.

Grey scale ultrasound imaging along with Doppler techniques are routinely used to aid in the diagnosis of CLD and portal hypertension. However, these techniques detect only morphological changes in the liver along with alteration in haemodynamics. Ultrasound elastography is a recent technique, which allows evaluation of tissue elasticity and hence, has an important role in evaluation of diseases such as CLD, which are associated with a change in tissue stiffness. Elastography can be broadly categorised as strain and Shear Wave Elastography. The latter is more useful for deep-seated structures and is of three types, including, 1D-Transient elastography (TE), point Shear Wave Elastography (pSWE) or Acoustic Radiation Force Impulse (ARFI) elastography, two-dimensional Shear Wave Elastography (2D-SWE). Although liver elastography is a good non-invasive method to detect liver stiffness (LS), biopsy is still the gold standard. For this reason, LS measured by elastography has made its way into the guidelines for diagnosis, stratification and classification of liver diseases. Several studies have been conducted correlating LS with portal hypertension and predicting the presence of EV.

Anatomically speaking, the splenic vein drains into the superior mesenteric vein to form the portal vein. Hence, it is directly connected to the portal vein. As a consequence, it can be presumed that any pathology affecting the blood flow in the portal vein can also affect the spleen. Many centres have focused on the use of splenic elastography in patients with liver disease, correlating splenic stiffness (SS) values with the stage of liver fibrosis.^6^ The spleen of cirrhotic patients is characterised by the presence of passive congestion, fibrotic hyperplasia, hyperactivated lymphoid tissue and angiogenesis. In the last few years, several studies have attempted to show that ultrasound based elastography using SS as a surrogate marker could accurately predict the presence of significant EV.

This study aimed to find any association between splenic shear wave velocity (SWV) measured by ARFI elastography and the presence of EV.

## Materials and methods

This quasi-experimental study design included 50 patients over 18 years of age with CLD who were undergoing screening endoscopic examination and were referred for upper abdominal ultrasonography. Fifty subjects who presented to the department of radiology for ultrasonography for an unrelated pathology were also included in the study as the control group.

Exclusion criteria for the patient group were: patients with a history of varices, any hepatic or splenic surgical procedure, focal hepatic pathologies, focal or diffuse splenic pathologies except for the presence of Gamna–Gandy bodies, and pregnancy. Exclusion criteria for the control group were: past history of jaundice or hepatitis, incidentally detected splenic or hepato-biliary pathology on upper abdominal ultrasonography, history of substantial alcohol intake (> 30 g/daily for men, > 20 g/daily for women),^7^ any prior surgical procedure on the spleen or liver in the past, and pregnancy.

The study was conducted at our institute over a duration of 12 months from 01 September 2018 to 31 August 2019 after obtaining ethical clearance from the institutional ethical committee. The procedure was explained to the patients and written informed consent was obtained. Demographics of the study subjects such as age and sex were recorded.

The control group as well as the patient group underwent routine grey scale upper abdominal ultrasonography on the Siemens Acuson S2000^TM^ ultrasound system (Siemens Medical Solutions, Mountain View, California, United States [US]) using a curvilinear array transducer (3 MHz–5 MHz) and a high frequency probe (5–17 MHz) for detailed evaluation when required.

**FIGURE 1:**
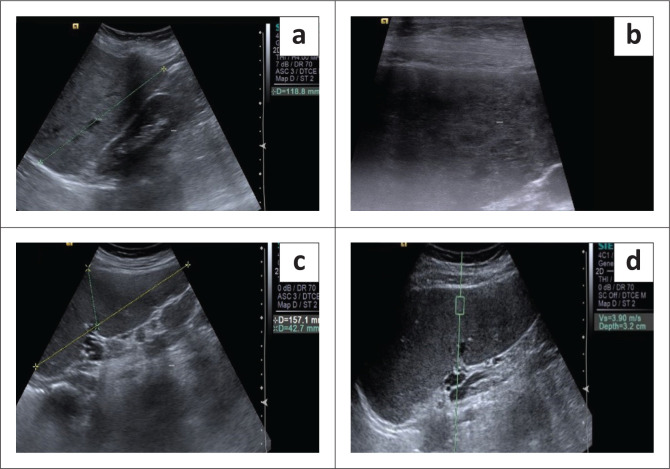
A 40-year-old female with chronic liver disease and oesophageal varices: Grey scale ultrasound reveals a normal sized liver with a heterogenous echo-pattern (a). Hepatic surface nodularity and echo-pattern is better appreciated with the high frequency probe (b). Splenomegaly with normal echo texture is seen (c). Acoustic radiation force imaging measurement taken in the splenic parenchyma is displayed in (d).

**FIGURE 2:**
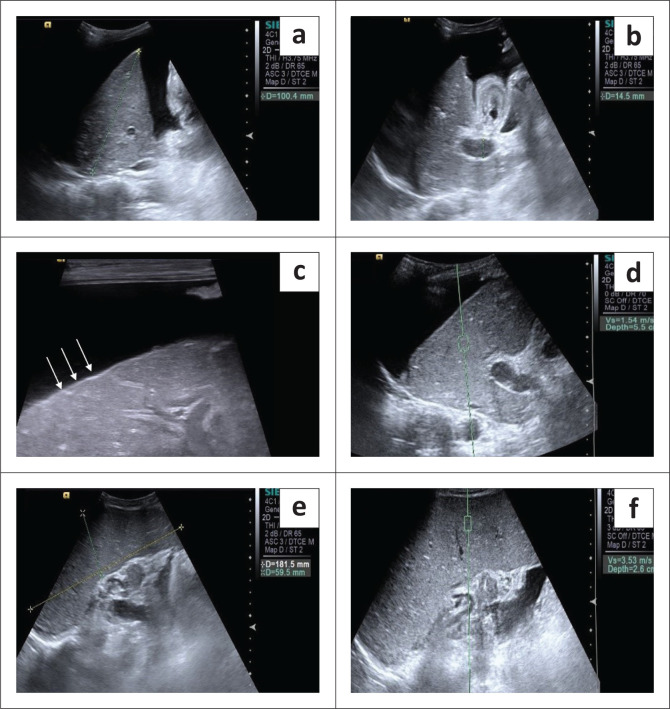
A 40-year-old female with chronic liver disease and large oesophageal varices: Grey scale ultrasound reveals gross ascites and an altered hepatic echo-pattern (a) with a dilated portal vein, measuring 14.5 mm at the porta and an oedematous gall bladder wall (b). Evaluation with the high frequency probe better depicts the hepatic surface nodularity (arrows) (c) Hepatic shear wave velocity (SWV) measures 1.54 m/s (d). Splenomegaly with normal echo texture (e). Splenic acoustic radiation force imaging measurement with ROI placed about 2.6 cm below the skin surface shows SWV in this case to be 3.53 m/s (f).

**FIGURE 3:**
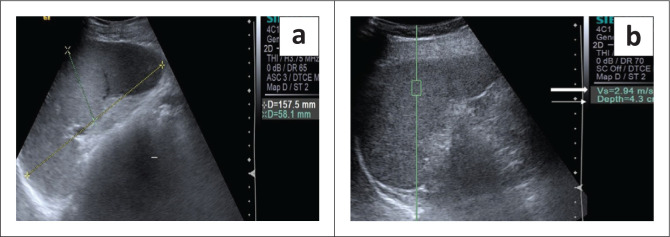
Splenic acoustic radiation force imaging (ARFI) evaluation in a control subject: Grey scale ultrasound (a) shows splenic longitudinal dimension taken as the maximum distance from upper to the lower pole at the level of splenic hilum (15.7 cm). (b) Shows ROI placement for splenic ARFI evaluation. The rectangular ROI was placed at a depth of 4.3 cm (arrow) from the skin surface in the splenic parenchyma devoid of blood vessels. Shear wave velocity (SWV) in this case was 2.94 m/s (solid arrow). Ten random SWV (in m/s) measurements were taken from the upper pole to the lower pole of the spleen during suspended respiration.

Liver size was measured as the maximum craniocaudal dimension in the midclavicular line. In addition, the presence of surface nodularity, caudate lobe hypertrophy and ascites was observed in the patient group (Figure 1 and Figure 2). Splenic bipolar diameter/longitudinal size was taken as maximum craniocaudal length at the level of splenic hilum. Splenic transverse diameter was taken in a plane orthogonal to the bipolar diameter (Figure 3). Splenic area was taken as the product of bipolar and transverse diameter as suggested by Loftus et al.^8^ and Ioanitescu ES^9^ in both the patient and control groups.

**FIGURE 4:**
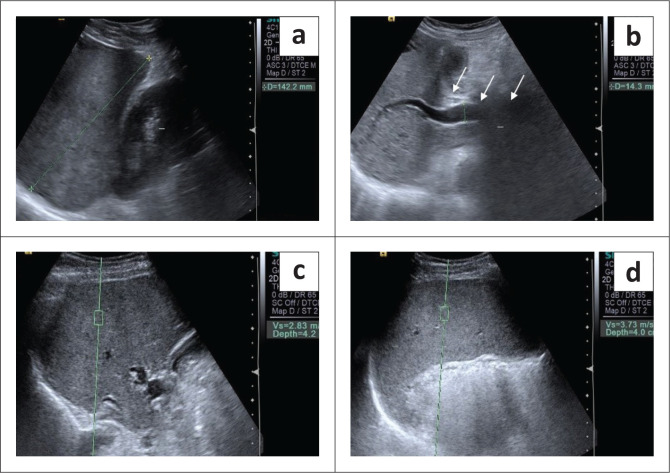
A 39-year-old male with chronic liver disease and oesophageal varices: Grey scale ultrasound reveals a normal sized liver with an altered echo-pattern (a). The portal vein is dilated, measuring 14.3 mm at the porta (b). Hepatic shear wave velocity (SWV) in the liver measures 2.83 m/s (c). Splenic acoustic radiation force imaging measurement shows SWV in this case to be 3.73 m/s (d).

Following grey scale ultrasound examination, splenic and LS were evaluated using the ARFI based elastography technique in the patient and control groups:

- Liver stiffness was evaluated using the ARFI enabled curvilinear transducer. A subcostal approach was used with subjects lying in the supine position and the right arm in maximum abduction behind the head. The scan was performed after 6 h of fasting and during the acquisition of SWV, the subjects were requested to hold their breath for 5 s. A region of interest (ROI) (10 mm^2^ × 6 mm^2^) was placed in the right lobe of liver at least 1 cm – 2 cm below the liver capsule (Figure 4). Special attention was paid to avoid any vessels, and rib shadows or artefacts from nearby lung, bowel or cardiac movements. Five SWV (in m/s) measurements were taken randomly throughout right lobe parenchyma. The mean and median values were calculated for both the patient and control groups.
- Splenic stiffness was measured with subjects lying in the right lateral decubitus position with the left arm in maximum abduction in order to increase the intercostal acoustic window. The procedure was similar to liver elastography with the scan performed in breath hold. The ROI was placed in the splenic parenchyma at least 1 cm – 2 cm below the splenic capsule (Figure 1 to Figure 4). Ten SWV (in m/s) measurements were taken randomly throughout the splenic parenchyma. The mean and median values were calculated for the patient and control groups.

Patients with CLD underwent UGIE within 4 weeks of the ARFI evaluation. Endoscopic findings regarding the presence and type of EVs were recorded. Oesophageal varices were classified according to the international expert recommendations into three groups:

- Patients without EVs
- Patients with small EVs (diameter of less than 5 mm)
- Patients with large varices (diameter greater than 5 mm)

Correlation of hepatic size, splenic size and splenic area was performed between (1) the patient and control groups and (2) patients without and with varices. Hepatic and splenic SWV were similarly compared between these two groups. Receiver operating characteristic (ROC) curves were drawn to obtain the cut-off values of hepatic and splenic SWV to predict the presence of EVs in patients with CLD.

### Statistical analysis

The collected data were transformed into variables, coded and entered in Microsoft Excel. Data were analysed and statistically evaluated using Statistical Package for Social Sciences (SPSS)-PC-25 version. The Kolmogorov–Smirnov test was used to see the normality distribution of data. Quantitative data were expressed as mean, standard deviation and the difference between two comparable groups, tested by the student’s *t*-test (unpaired) or Mann–Whitney ‘U’ test, while qualitative data were expressed as percentages. A *p*-value less than 0.05 was considered statistically significant. Receiver operating characteristic curves were drawn to determine the cut-off values for splenic length, splenic area, hepatic and splenic SWV based on which sensitivity, specificity, positive predictive value (PPV) and negative predictive values (NPV) were calculated. The Spearman’s rank correlation coefficient (non-parametric) was used to determine correlations between different quantitative variables.

### Ethical considerations

The study was conducted at our institution after obtaining ethical clearance from the Institutional Ethical Committee of Maulana Azad Medical College (F.No.17/IEC/MAMC/2018/15) 26 October 2018.

## Results

The final study sample included 100 participants. The 50 volunteers in the control group had a mean age of 32.56 ± 12.74, and a median age of 28 years. There were 26 (52%) males and 24 (48%) females in this group. The mean and median age of the 50 participants in the patient group was 41.36 ± 13.39 and 42 years, respectively. There were 30 (60%) males and 20 (40%) females in the patient group.

The liver size, splenic size and splenic area measurements in both the groups are presented in Table 1. While liver size was quite similar in the two groups, splenic size and splenic area were higher in the patient group and this difference was found to be statistically significant (*p* < 0.001).

**TABLE 1:** Comparison of various parameters in the control group and patient group (*N* = 100).

Parameters	Control group (*n* = 50)		Patient group (*n* = 50)			*p*
	Mean ± s.d.	Range	Mean ± s.d.	Range	%	
**Grey scale ultrasonography**						0.97
Liver size (in cm)	12.19 ± 1.69	8.8 cm – 15.3 cm	12.13 ± 2.05	6.8 m/s – 15.8 m/s	-	-
Altered hepatic echotexture	Nil	Nil	43	-	86	-
Nodular hepatic surface	Nil	Nil	39	-	78	-
Caudate lobe hypertrophy	Nil	Nil	7	-	14	-
Ascites	Nil	Nil	12	-	24	-
Porto-systemic collaterals	Nil	Nil	8	-	16	-
Splenic size (in cm)	9.52 ± 1.55	6.8 cm – 12.2 cm	14.17 ± 3.51	8.1 cm – 25.3 cm	-	< 0.001
Splenic area (in cm^2^)	37.47 ± 12.95	-	86.64 ± 53.19	-	-	< 0.001
**Shear wave elastography**						
Hepatic SWV (in m/s)	1.22 ± 0.25	0.72 m/s – 2.0 m/s	2.37 ± 0.62	0.72 m/s – 3.65 m/s	-	< 0.001
Splenic SWV (in m/s)	2.21 ± 0.30	1.48 m/s – 2.72 m/s	3.33 ± 0.44	2.44 m/s – 4.33 m/s	-	< 0.001

s.d., standard deviation; SWV, shear wave velocity.

The elastography findings of the liver and spleen in both groups are also presented in Table 1. Both the hepatic and splenic SWVs were significantly higher in the patient group than in the control group with *p* < 0.001.

On calculation of Spearman’s correlation coefficient for the control group, hepatic SWV appeared to have no significant correlation with age (*r* = 0.179) or liver size (*r* = 0.025). Similarly, no significant correlation of splenic SWV with age (*r* = 0.482), splenic size (*r* = 0.441) or with splenic area (*r* = 0.394) was noticed. In the patient group, hepatic SWV was found to have no significant correlation with age (*r* = 0.115, *p* = 0.426) or liver size (*r* = 0.025). On calculation of Spearman’s correlation coefficient, splenic SWV was found to have no significant correlation with age (*r* = 0.00), splenic size (*r* = 0.191) or with splenic area (*r* = 0.253).

Patients underwent UGIE within 4 weeks of the ARFI evaluation. Upon endoscopic evaluation, it was found that 10/50 patients (20%), had no EVs and 40/50 patients (80%), showed evidence of either small or large EVs, 20 in each category. Table 2 displays the important differences in grey scale and SWV findings in patients with and without varices.

**TABLE 2:** Comparison of various parameters between patients with varices and without varices (*n* = 50).

Parameters	Patients without varices (*n* = 10) (mean ± s.d.)	Patients with varices (*n* = 40) (mean ± s.d.)	*p*
Grey scale ultrasonography			
Liver size (in cm)	12.06 ± 2.49	12.15 ± 1.96	0.96
Splenic size (in cm)	12.20 ± 1.97	14.67 ± 3.65	0.04
Splenic area (in cm^2^)	65.08 ± 32.94	92.03 ± 56.17	0.15
Shear wave elastography			
Hepatic SWV (in m/s)	2.41 ± 0.38	2.36 ± 0.67	0.77
Splenic SWV (in m/s)	2.87 ± 0.19	3.451 ± 0.41	< 0.01

s.d., standard deviation; SWV, shear wave velocity.

Both the liver size and hepatic SWV were quite similar between the two subgroups. While both the splenic size and splenic area were higher in the patients with varices, this difference achieved statistical significance only for the splenic size. There was a statistically significant difference in the splenic SWV between the two groups (*p* < 0.01).

## Discussion

The idea of a non-invasive radiological investigation that allows for the assessment of patients with a high risk of developing varices, and consequently limits the number of patients undergoing invasive UGIE, is very attractive. Previous studies have correlated various radiological and laboratory parameters, either alone or in combination, with the presence or absence of EVs in patients with CLD.

The use of non-invasive techniques such as elastography for the evaluation of LS and of late, SS, has become popular. Recent literature has also described their usefulness in the assessment of CLD and prediction of risk for EVs.^5,6,10,11,12,13,14,15,16,17,18,19,20^ Elastography techniques can be classified as strain imaging (SI) and shear wave imaging (SWI) depending on the measured physical quantity. A normal stress is applied to tissue and the normal strain is measured in SI whereas a dynamic stress is applied to tissue by using a mechanical vibrating device or acoustic radiation force in SWI. Shear wave elastography determines the mechanical properties of tissues by monitoring the speed of shear waves generated by the ultrasound-induced acoustic radiation force.^12^

Shear wave elastography allows real-time visualisation of the organ, allowing accurate placement of the ROI for measurement of elastographic values. Another advantage is that it can be performed during routine ultrasonographic examination of the hepatobiliary system and hence can be easily incorporated in the routine follow-up of patients with cirrhosis. In this study we assessed the SS in patients of CLD and its association with EV using pSWE.

On comparing the grey scale ultrasonographic findings between the patient and control groups, we found that both groups were similar in terms of mean hepatic size. The patient group, however, had a statistically significant larger splenic size (14.17 ± 3.51 vs 12.20 ± 1.97) and splenic area (86.64 ± 53.19 vs 37.47 cm^2^ ± 12.95 cm^2^) compared with control group (*p* < 0.001). Although initially splenic enlargement in patients with CLD was largely attributed to portal congestion, several studies have demonstrated that the pathophysiology is more complex, and splenomegaly has a congestive and a hyperplastic component.^21^ Increased activation of the mammalian target of rapamycin (mTOR) signalling pathway has also been observed as a cause of splenomegaly according to one of the studies.^22^

The mean hepatic size was similar in patients with and without varices (12.15 cm ± 1.96 cm vs 12.06 cm ± 2.49 cm). This is in contrast to the findings of a previous study by Nouh et al.,^23^ who found the liver to be shrunken in patients with varices with the mean right lobe of the liver spanning 12.63 cm ± 1.16 cm and 14.04 cm ± 1.13 cm in patients with and without varices, respectively. This discrepancy could be because of the difference in sample sizes between the two studies as the authors evaluated a larger number of patients (*n* = 200) as well as a larger number of patients with advanced cirrhosis, with 54.5% of patients having bleeding EV. This contrasts with the current study where patients displayed varying grades of CLD. Sharma and Aggarwal also reported similar findings in their study;^24^ however, their patients displayed relatively severe disease with ascites and a history of hepatic encephalopathy in 80% and 39% of subjects, respectively, compared with 24% patients with ascites in this study and none with a history of hepatic encephalopathy.

The mean splenic size was significantly higher in patients with varices than in those without varices (14.67 cm ± 3.65 cm vs 12.20 cm ± 1.97 cm, *p* = 0.04). This finding concurs with previous studies that have also reported significantly larger splenic sizes in patients with varices than in those without varices.^25,26,27^ Even though the mean splenic area too was larger in patients with varices than in those without varices, no statistically significant difference was observed between the subgroups (*p* = 0.15). Using a splenic length cut-off value of 15.1 cm for the prediction of EV in the patients of CLD, we obtained a specificity (Sp) of 100%, although the sensitivity (Sn) was only 50%.

Ultrasound elastography assessment of the liver and spleen revealed that patients with CLD had significantly higher SWV values for both organs as compared with the controls (*p* < 0.001). These findings are in concordance with the outcomes of a previous study,^28^ in which an alteration in the elasticity of the hepatic and splenic parenchyma was observed in patients with CLD with the LS measuring 2.50 m/s ± 0.80 m/s and 0.91 m/s ± 0.29 m/s in patients and controls, respectively, (*p* < 0.001). The SS was also significantly higher among patients compared with controls (3.14 ± 0.80 vs 1.89 ± 0.26, respectively, *p* < 0.001). Although the exact values in the current study were higher, the general trend was similar in both studies.

There was no statistically significant correlation between the hepatic SWV of patients with and without varices (2.41 m/s ± 0.38 m/s v. 2.36 ± 0.67, *p* = 0.77) consistent with the findings of some of the previous studies.^29,30^ However, there have been studies that have found a correlation between LS and the presence and severity of EVs.^31,32^ Similarly, some authors also found that LS can predict the presence of poor prognostic factors such as EV in patients with liver cirrhosis.^27,33^

**FIGURE 5:**
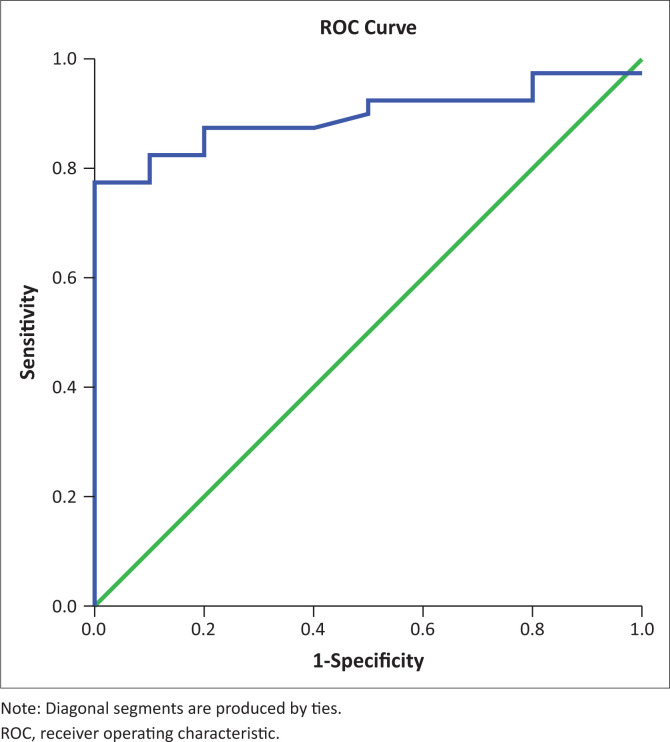
Receiver operating characteristic curve of splenic shear wave velocity for the prediction of oesophageal varices in patients of chronic liver disease.

**FIGURE 6:**
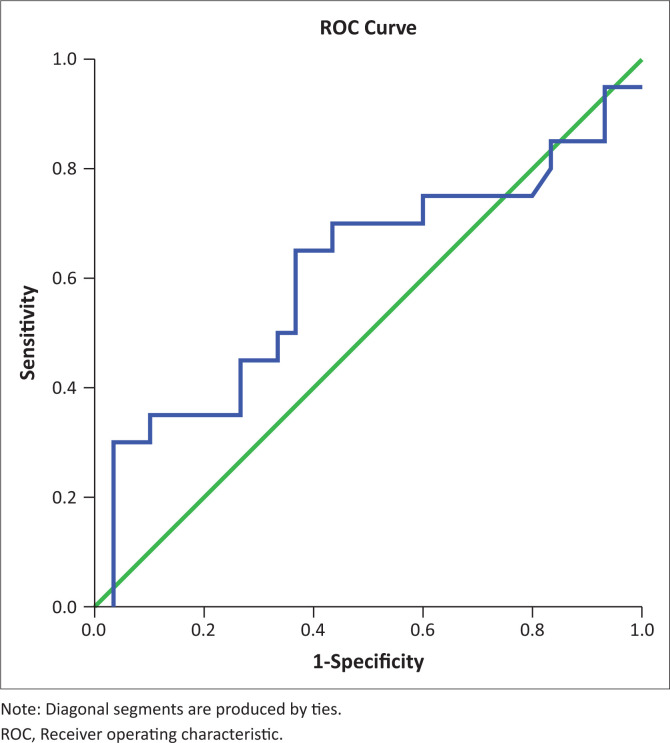
Receiver operating characteristic curve of spleen shear wave velocity for the prediction of large oesophageal varices in patients of chronic liver disease.

On the other hand, the mean splenic SWV in patients with varices was significantly higher than in patients without varices (3.451 m/s ± 0.41 m/s vs 2.87 m/s ± 0.19 m/s, *p* < 0.01). This is consistent with the findings of several previous studies.^33,34,35,36,37,38^ Park Y et al., in their study found the mean SWVs to be 2.39 m/s ± 0.67 m/s, 2.19 m/s ± 0.73 m/s and 1.64 m/s ± 0.57 m/s, respectively, in patients with high risk, low risk and no EVs.^37^ Although the absolute values are lower than in this study, the general trend is similar between the two studies. The ROC curves of splenic SWV for the prediction of EVs in patients with CLD have been depicted in Figure 5 and Figure 6. This emphasises the non-invasive role of splenic elastography in the prediction of EVs. As the number of patients in each group was relatively small, we did not attempt to compare the findings between patients with small and large varices.

The authors attempted to determine cut-off values for elastographic parameters such as hepatic SWV, splenic SWV and a combination of the same to determine the presence of varices using ROC curves. In this study, a cut-off value of 2.608 m/s was obtained for hepatic SWV with a sensitivity (Sn) of 37.5%, specificity (Sp) of 80%, NPV of 24.2% and PPV of 88.3% for prediction of EV in patients with an accuracy of 47%. Similarly, a cut-off value of 3.13 m/s was obtained for splenic SWV with a Sn of 77.5%, Sp of 100%, NPV of 52.6% and PPV of 100% for prediction of EV in patients with an accuracy of 89%. The area under curve (AUC) was much greater for splenic SWV than hepatic SWV (0.89 vs. 0.47), confirming higher accuracy of splenic SWV in predicting EV in patients with CLD. This was in concordance with the meta-analysis performed by Manatsathit W et al.,^39^ who reported a Sn of 90%, Sp of 73% for the splenic SWV and inferred that SS was superior to LS (AUC 0.89 vs. 0.85) for the detection of EV. Furthermore, AUC was greater for the combination of splenic and hepatic SWV than splenic SWV alone with a combined Sn of 82.5%, Sp of 100%, PPV 100%, NPV 58.8% suggesting that it is even more accurate for the prediction of EV. Thus, use of this combination may be superior to splenic SWV alone for prediction of EVs.

This study had a few limitations including the relatively small sample size. Although we included 50 patients, the number of patients included in each sub-group, and the number of patients with and without varices, and with small and large varices, were relatively small. Hence, we could not assess the usefulness of splenic elastography in patients with small and large EVs. Further studies with larger number of patients may help in the validation of this parameter as a predictor of large EV. Additionally, the control group, although equal in number to the patient group, was not age and sex matched.

## Conclusion

Chronic liver disease causes a significant increase in the stiffness of the liver and spleen with SWV values for the spleen being higher for patients with varices emphasising the role of elastography as a non-invasive predictor for the presence of EVs. Among all the individual radiological parameters evaluated in this study, splenic SWV had the highest sensitivity and specificity, which was further augmented by a combination of hepatic and splenic SWV. This study found that splenic SWV alone or in combination with hepatic SWV is a useful non-invasive technique for the prediction of presence of EVs.
